# Acute Chagas Disease Induces Cerebral Microvasculopathy in Mice

**DOI:** 10.1371/journal.pntd.0002998

**Published:** 2014-07-10

**Authors:** Lindice Mitie Nisimura, Vanessa Estato, Elen Mello de Souza, Patricia A. Reis, Marcos Adriano Lessa, Hugo Caire Castro-Faria-Neto, Mirian Claudia de Souza Pereira, Eduardo Tibiriçá, Luciana Ribeiro Garzoni

**Affiliations:** 1 Laboratório de Investigação Cardiovascular, Instituto Oswaldo Cruz, Fundação Oswaldo Cruz, Rio de Janeiro, Rio de Janeiro, Brazil; 2 Laboratório de Morfologia e Morfogênese Viral, Instituto Oswaldo Cruz, Fundação Oswaldo Cruz, Rio de Janeiro, Rio de Janeiro, Brazil; 3 Laboratório de Imunofarmacologia, Instituto Oswaldo Cruz, Fundação Oswaldo Cruz, Rio de Janeiro, Rio de Janeiro, Brazil; 4 Laboratório de Ultra-estrutura Celular, Instituto Oswaldo Cruz, Fundação Oswaldo Cruz, Rio de Janeiro, Rio de Janeiro, Brazil; Albert Einstein College of Medicine, United States of America

## Abstract

Cardiomyopathy is the main clinical form of Chagas disease (CD); however, cerebral manifestations, such as meningoencephalitis, ischemic stroke and cognitive impairment, can also occur. The aim of the present study was to investigate functional microvascular alterations and oxidative stress in the brain of mice in acute CD. Acute CD was induced in Swiss Webster mice (SWM) with the Y strain of *Trypanosoma cruzi* (*T. cruzi*). Cerebral functional capillary density (the number of spontaneously perfused capillaries), leukocyte rolling and adhesion and the microvascular endothelial-dependent response were analyzed over a period of fifteen days using intravital video-microscopy. We also evaluated cerebral oxidative stress with the thiobarbituric acid reactive species TBARS method. Compared with the non-infected group, acute CD significantly induced cerebral functional microvascular alterations, including (i) functional capillary rarefaction, (ii) increased leukocyte rolling and adhesion, (iii) the formation of microvascular platelet-leukocyte aggregates, and (iv) alteration of the endothelial response to acetylcholine. Moreover, cerebral oxidative stress increased in infected animals. We concluded that acute CD in mice induced cerebral microvasculopathy, characterized by a reduced incidence of perfused capillaries, a high number of microvascular platelet-leukocyte aggregates, a marked increase in leukocyte-endothelium interactions and brain arteriolar endothelial dysfunction associated with oxidative stress. These results suggest the involvement of cerebral microcirculation alterations in the neurological manifestations of CD.

## Author Summary

Chagas disease (CD) is a neglected tropical illness caused by the parasite *Trypanosoma cruz*i (*T. cruzi*). It is endemic in Latin America and affects 10 million people worldwide. Meningoencephalitis occurs in children with acute CD and in immunosuppressed patients suffering acute CD reactivation. During the chronic phase, cerebral manifestations, including ischemic stroke and cognitive impairment, can also occur. Although microvascular alterations have been implicated in Chagas cardiomyopathy, the main clinical form of the disease, there is a lack of discussion in some studies regarding alterations of the cerebral microcirculation in CD. In the present study, we evaluated the functionality of the cerebral microcirculation in mice infected by *T. cruzi*. Utilizing an intravital video-microscope, we observed in the brain of infected mice a reduction in the number of perfused capillaries, an increased interaction between inflammatory cells and venules, the presence of microvascular platelet-leukocyte aggregates and alterations in the dilatation capacity of arterioles. Moreover, cerebral oxidative stress was increased in infected animals. We concluded that acute CD induced cerebral microvasculopathy.

## Introduction

Chagas disease (CD), which is caused by the protozoan *Trypanosoma cruzi* (*T. cruzi*), is endemic in Latin America and affects approximately 10 million people worldwide [1]. Cardiomyopathy is the main clinical manifestation of CD, but digestive and neurological forms can also occur [2]. Meningoencephalitis is an important manifestation of acute CD in children under 2 years of age [3], and it is also frequently observed in immunosuppressed patients suffering from acute CD reactivation [4]. Ischemic stroke is the main neurological manifestation observed in chronic CD [5], and cognitive impairment and depression can also occur [6], [7]. Moreover, experimental studies in mice have shown that depressive-like behavior is independent of central nervous system inflammation but is associated with high levels of systemic tumor necrosis factor (TNF) [8].

Acute CD has re-emerged in oral transmission outbreaks in countries where vector transmission has been controlled [9]. During acute CD, the peripheral inflammatory response is characterized by the presence of macrophages [10], NK cells [11] and intense lymphocyte polyclonal activation [12]. This response is followed by the systemic synthesis of pro-inflammatory cytokines [13], nitric oxide (NO) [14] and reactive oxygen species [15]. Microvascular alterations have been implicated in the pathogenesis of Chagas cardiomyopathy and include vascular constrictions, microaneurysms, dilatations and platelet aggregation, resulting in the formation of transient occlusive thrombi. These alterations contribute to myocytolytic necrosis followed by inflammatory infiltration and interstitial fibrosis. Moreover, vasoactive substances, including endothelin-1 and thromboxane, are involved in the modulation of vascular responses during *T. cruzi* infection, contributing to platelet aggregation, microvascular spasms and endothelial dysfunction [16].

Using intravital video-microscopy (IM), our research group recently demonstrated that cerebral functional microvascular alterations are pathophysiologically relevant in models of systemic severe infectious syndromes, such as sepsis and malaria, in mice [17], [18]. Moreover, IM has been used as an important tool with which to evaluate the microcirculation during *T. cruzi* infection, e.g., using the hamster cheek pouch and cremaster muscle models [19]–[21].

In experimental models of CD, despite reports that inflammatory cells migrate to the cerebral tissue in a VLA-4^+^-VCAM-1-dependent manner [22] and that *T. cruzi* infects cerebral endothelial cells [23], no studies have directly characterized the functional brain microcirculation during *T. cruzi* infection. In this paper, we present results from analyses of the consequences of acute CD on cerebral microcirculation in mice. We present evidence that acute infection by *T. cruzi* increases oxidative stress in the brain and causes severe cerebral vasculopathy, which may contribute to the neurological manifestations of CD.

## Methods

### Ethics statement

All procedures were approved by the Oswaldo Cruz Foundation Animal Welfare Committee (License numbers LW-40/13 and LW-74/12) and were consistent with the USA National Institutes of Health Guide for the Care and Use of Laboratory Animals (NIH Publication No. 85-23, revised 1996).

### Animals

We used outbred male Swiss Webster mice (SWM) (age 6 to 8 weeks), weight 18 to 20 g) obtained from the Oswaldo Cruz Foundation Animal Facilities (CECAL, Rio de Janeiro, Brazil). The animals were housed for at least 1 week before parasite infection under conditions of controlled light (12∶12 h light-dark cycle) and temperature (22±1°C).

### Experimental groups

The mice were randomly divided into two groups: a non-infected (NI) control group (n = 5/experiment) and a *T. cruzi* (Y strain)-infected experimental group (n = 15/experiment). Infection was performed by intraperitoneal injection of 10^4^ bloodstream trypomastigote forms of *T. cruzi*. Age-matched, non-infected mice were maintained under identical conditions. Two to three independent experiments were performed depending on the procedure.

### Parasitemia, body weight and mortality

Parasitemia was individually assessed using the Pizzi-Brener method by direct microscopic counting of parasites in 5 µl of tail blood. Body weight and mortality were regularly monitored for twenty-two days post-infection (dpi) in three independent experiments (n = 15 animals/experiment).

### Intravital video-microscopy in the brains of mice

We anesthetized animals from the NI control and *T. cruzi*-infected groups at 8 and 15 dpi by intraperitoneal injection with a mixture of xylazine (10 mg/kg) and ketamine hydrochloride (75 mg/kg). The animals were tracheostomized and artificially ventilated with room air. We cannulated the jugular vein to allow the injection of fluorescent tracers. Body temperature was maintained at 37°C with a homeothermic blanket system. The animals were immobilized in a stereotaxic frame, and a cranial window was created by craniotomy with a high-speed drill to expose the cerebral microcirculation [24]. The animals were then placed on an upright fixed-stage of an intravital microscope with a mercury lamp (Olympus BX51/WI, USA) attached to a CCD digital video camera system. The microscopic field was continuously superfused with artificial cerebrospinal fluid at 37°C, pH 7.35 by an infusion pump (Harvard apparatus plus, USA) connected to catheters fixed over the opened cranial window. The superfusate was continuously aerated with 10% O_2_, 6% CO_2_ and 84% N_2_ to maintain tension and a gas composition comparable to physiological pH and to avoid local inflammation. We performed two independent experiments with IM (n = 4 animals/experiment).

### Assessment of functional capillary density

After the intravenous administration of 0.1 mL of 5% FITC-labeled dextran, microscopic images of the cerebral microcirculation were acquired by Archimed 3.7.0 software (Microvision, Evry, France) for online counting of capillaries using Saisam software (Microvision, Evry, France). The functional capillary density, or the total number of spontaneously perfused capillaries (i.e., vessels with diameters less than 10 µm) per square mm of surface area (1 mm^2^), was determined by counting each capillary branch in 4 fields over a period of 4 minutes, as previously described in detail [25]. The capillaries measured approximately 5 to 10 µm in diameter, connected arterioles to venules and contained a single column flow of red blood cells [26]. These cells are biconcave-shaped cells with highly deformable membranes, which allows the cells to traverse narrow passages with small diameters (e.g., capillaries) [26].

### Leukocyte rolling and adhesion analysis

We labeled circulating leukocytes by injecting the mice with intravenous rhodamine-6G (0.3 mg/kg), which also stained circulating platelets [17]. The fluorescent leukocytes were made visible by epi-illumination through the cranial window. We observed five randomly selected venular segments (30 to 100 µm in diameter and 100 µm long) for 60 seconds in each preparation. Leukocyte-endothelial interactions were evaluated by determining the number of (i) rolling leukocytes, defined as cells crossing the venular segment (100 µm) at a speed less than that of the circulating red blood cells (presented as the number of cells/min/100 µm), and (ii) leukocytes that adhered for at least 30 seconds to the venular wall. Considering that rhodamine-6G stained both platelets and leukocytes, we also investigated the percentage of microvessels exhibiting platelet-leukocyte aggregates (PLAs) [27] in five microscopic fields per animal.

### Assessment of oxidative stress

To characterize the oxidative stress in the brains of mice, we measured levels of thiobarbituric acid reactive species (TBARS) [28]. The brains of NI and *T. cruzi*-infected mice were homogenized in a cold phosphate buffer, pH 7.4, with 2,6-bis(1,1-dimethylethyl)-4-methylphenol (BHT, final concentration 0.2%). Briefly, the samples (0.5 mL) were mixed with an equal volume of 0.67% thiobarbituric acid and then heated at 96°C for 30 min. The TBARS level was determined by the absorbance at 535 nm. Results are presented as malondialdehyde (ε = 1.56×10^5^ M^−1^ cm^−1^) per milligram of protein (BCA assay). We performed two independent experiments (n = 4 animals/experiment).

### Cerebral endothelium-dependent vasodilator responses

We evaluated vasodilator responses to the topical application of endothelium-dependent vasodilator acetylcholine (Ach; 10^−6^ M) in cerebral arterioles of both animal groups. The cranial window was suffused with Ach for five minutes, and the arteriolar diameters were measured before and after exposure to the vasoactive substance. Vascular responses are expressed as the percent (%) change from baseline.

### Statistical analysis

We expressed the results as the mean ± SEM for each group, and comparisons between groups were performed using unpaired *t*-tests or analysis of variance (ANOVA) followed by Bonferroni's multiple comparison test. Differences with *p* values of less than 0.05 were considered statistically significant. We used a commercially available, computer-based statistical package (GraphPad InStat 5.0, GraphPad Software Inc., La Jolla, CA, USA) for all calculations.

## Results

### Characterization of experimental acute Chagas disease in mice

**Figure 1:**
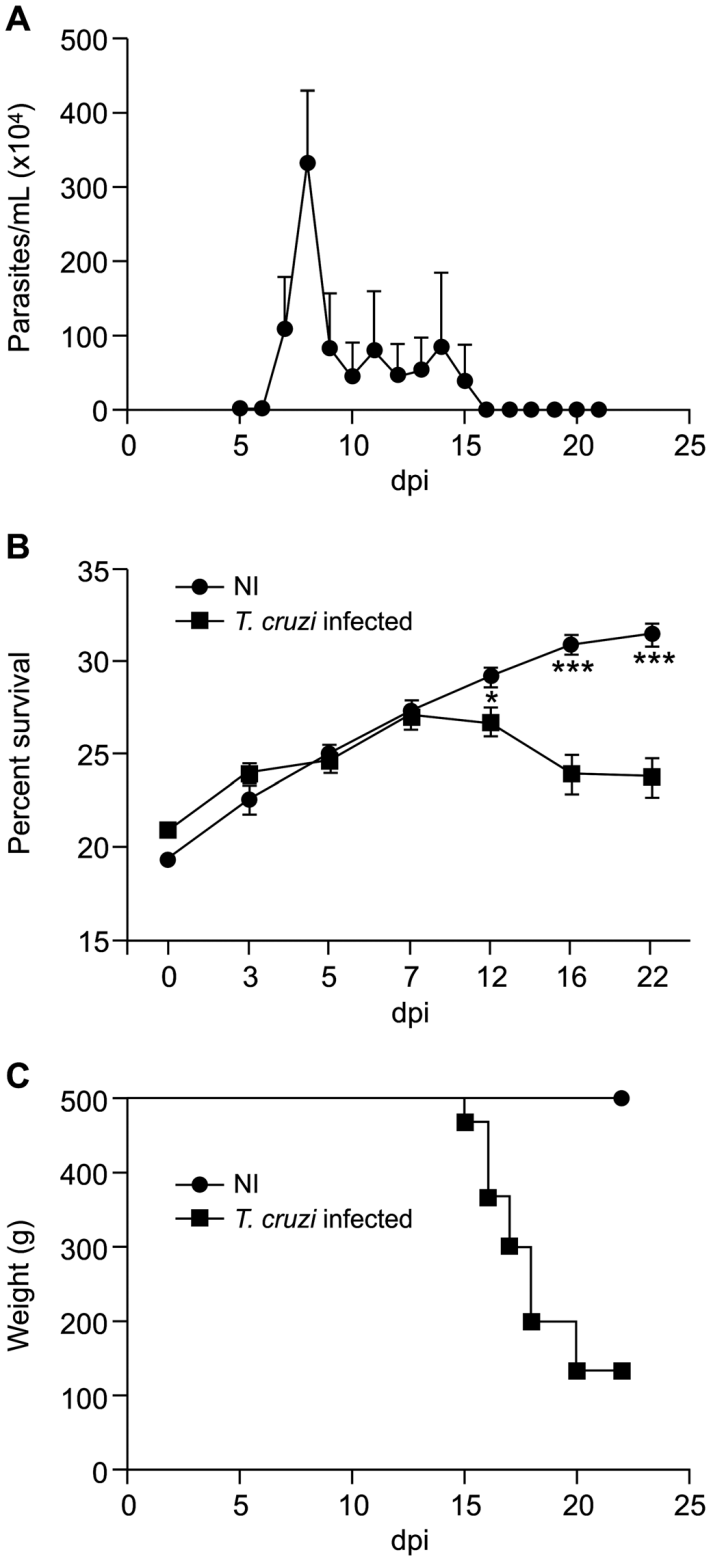
Swiss Webster mice infected with the Y strain of *T. cruzi*-developed acute CD. Mice were infected with 10^4^ blood trypomastigote forms, and the following parameters were evaluated in a kinetic study: (A) parasitemia, (B) weight and (C) survival rate. The parasitemia peak occurred at 8 dpi (A). *T. cruzi* infection induced a significant body weight decrease in a time-dependent manner, starting at 12 dpi. At 22 dpi, the average weight of the NI mice was 31.4±1.9 g, while that of the *T. cruzi*-infected group was 23.8±2.5 g (B). At 22 dpi, only 20% of the infected animals survived (C). Quantitative data are expressed as the means ± SEM (n = 20). One-way ANOVA test, *p*<0.05^*^ and *p*<0.001^***^, comparing the infected group at 15 dpi with the NI group; dpi: days post-infection; NI: non-infected.

The analysis of trypomastigote forms of *T. cruzi* in the blood of animals revealed that the peak of parasitemia occurred at 8 dpi (Figure 1A). At 8 dpi, *T. cruzi* infection induced significant changes in body weights in a time-dependent manner. At 22 dpi, the average weight of mice in the NI group was 31.4±1.9 g, while that of mice in the *T. cruzi*-infected group was 23.8±2.5 g (*p*<0.001; Figure 1B). Cardiac parasitism and inflammation were also observed at 15 and 22 dpi (data not shown). Only 20% of the infected animals survived to 22 dpi (Figure 1C).

### Acute *T. cruzi* infection induces changes in cerebral microvasculature perfusion

**Figure 2:**
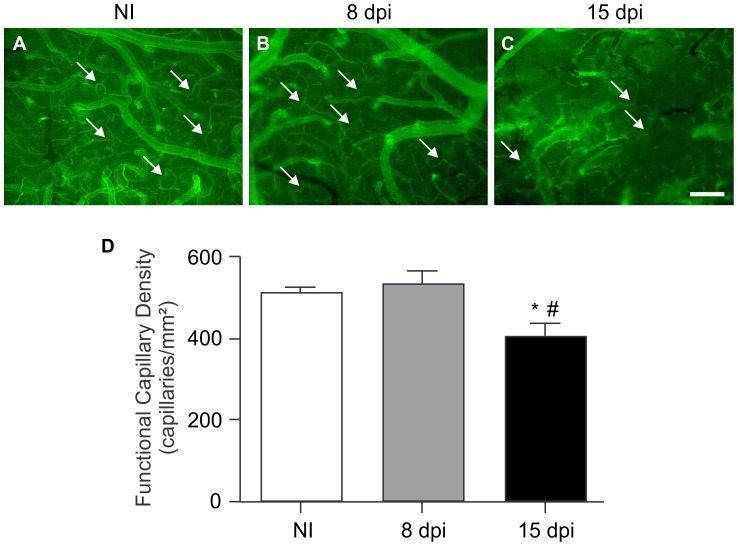
Acute CD causes cerebral functional capillary rarefaction. Perfused cerebral arterioles and capillaries (arrows) can be observed by the fluorescence of FITC-dextran in the non-infected (A) and *T. cruzi*-infected animals at 8 (B) and 15 (C) dpi. A collapse in the microcirculation can be observed at 15 dpi (C). In (D), the graph shows a significant reduction in the number of perfused blood vessels (capillary density) in the infected animals at 15 dpi (405±31.4 capillaries/mm^2^) compared with the non-infected controls (514±1 capillaries/mm^2^) and with the *T. cruzi*-infected mice at 8 dpi (535±31.2 capillaries/mm^2^). Quantitative data are expressed as means ± SEM (n = 5–8/group). One-way ANOVA test; bar = 100 µm; dpi: days post-infection; *p*<0.05: * comparing the infected animals at 15 dpi with NI group; # comparing 15 to 8 dpi. dpi: days post infection. NI: non-infected.

The visualization of FITC-labeled dextran by intravital video-microscopy revealed that at 15 dpi, *T. cruzi*-infected mice presented cerebral microcirculatory collapse, characterized by a significant change in the pattern of microvascular perfusion (Figure 2C) compared with the NI group (Figure 2A). This collapse was not observed in animals at 8 dpi (Figure 2B). The quantification of perfused capillaries showed that at 15 dpi, the infected group presented a significant reduction of perfused capillaries (405±31.4 capillaries/mm^2^) compared with the NI controls (514±11 capillaries/mm^2^; *p*<0.05) and the infected animals at 8 dpi (535±31.2 capillaries/mm^2^; *p*<0.01; Figure 2D).

### Cerebral leukocyte-endothelial interactions and brain oxidative stress increase during acute *T. cruzi* infection

**Figure 3:**
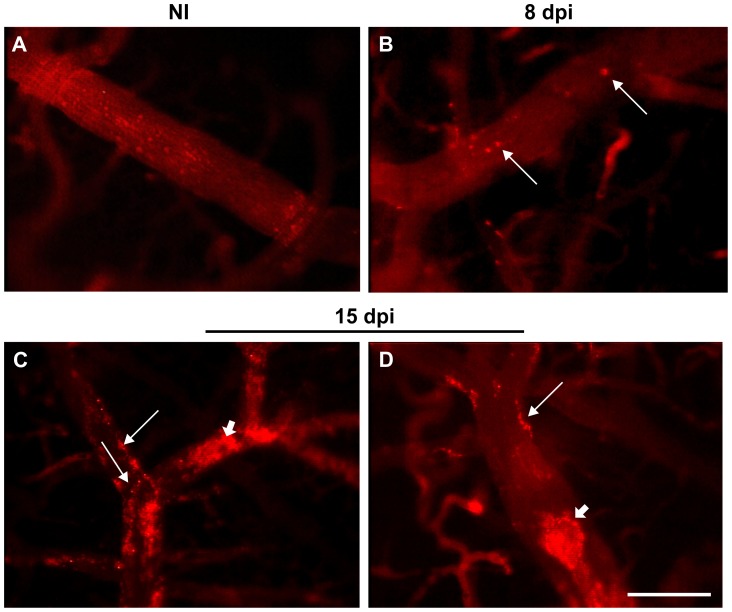
Rhodamine-labeled leukocytes in cerebral venules in acute CD. The images show venules of the non-infected (A) and *T. cruzi*-infected (B) mice at 8 and 15 dpi (C and D). The leukocyte-endothelium interaction (arrows) in the venules of the infected animals can be observed. Note the microvascular platelet-leukocyte aggregates (arrowhead) at 15 dpi in the infected animals (C and D). dpi: days post-infection; NI: non-infected.

The analysis of rhodamine-labeled leukocytes by intravital microscopy showed an increased number of leukocytes in the cerebral venular segment in *T. cruzi*-infected animals at both 8 (Figure 3B) and 15 (Figures 3C and D) dpi compared with the NI controls (Figure 3A). At 15 dpi, microvascular PLAs were present in a large number of venules (Figures 3C and D).

**Figure 4:**
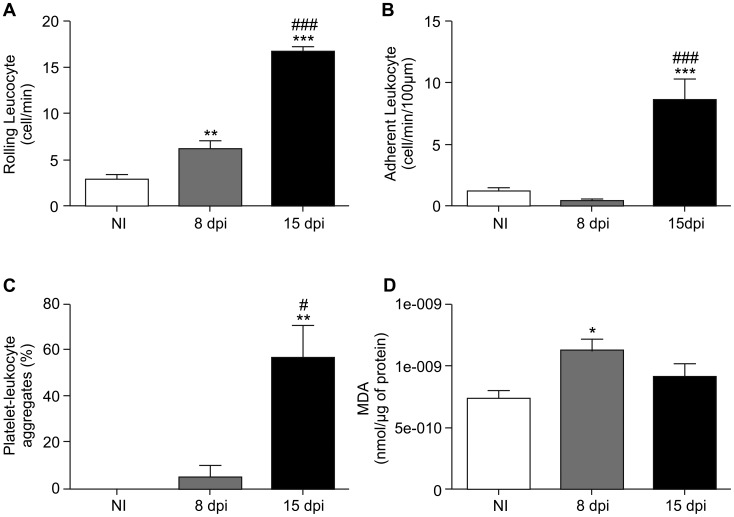
Acute CD increases the cerebral leukocyte-endothelium interaction, the percentage of microvessels presenting platelet-leukocyte aggregates and oxidative stress. An increased number of rolling cells was found in the cerebral venular segment of infected mice (A). The NI group presented only 3±0.5 cells/min. At 8 dpi, the animals presented 6.3±0.8 cells/min, and at 15 dpi, the number of rolling leukocytes increased to 16±0.6 cells/min. The leukocyte adhesion analysis (B) showed that 1±0.2 cells/min/100 µm adhered to the venular segment in the NI group. This number was 0.4±0.2 cells/min/100 µm at 8 dpi and 8.6±1.7 cells/min/100 µm at 15 dpi. At 15 dpi, a high percentage of venules showed microvascular platelet-leukocyte aggregates (PLAs) (C). The malondialdehyde (MDA) levels in the brain of the non-infected and *T. cruzi*-infected mice showed an increase in oxidative stress only at 8 dpi (D). A–C and H–K, bar = 100 µm. One-way ANOVA test; *p*<0.05^*^, *p*<0.01^**^and *p*<0.001^***^, comparing the infected animals at 15 dpi with the NI group; *p*<0.001^###^ and *p*<0.01^##^, comparing the infected animals at 15 and 8 dpi. Values are the means ± SEM (n = 4–8 animals/group); dpi: days post-infection; NI: non-infected.

The quantitative analysis showed 3±0.5 cells/min rolling in venules of NI animals (Figure 4A) and 6.3±0.8 cells/min rolling at 8 dpi (*p*<0.01). This number further increased to 16±0.6 cells/min at 15 dpi (*p*<0.001). Statistical analysis showed that the difference between 8 and 15 dpi was significant (*p*<0.001). As shown in Figure 4B, leukocyte adhesion in infected animals increased at 15 dpi (8.6±1.7 cells/min/100 µm) compared with the NI group (1±0.2 cells/min/100 µm, *p*<0.001) and with the values in the infected mice at 8 dpi (0.4±0.2 cells/min/100 µm, *p*<0.001). A high percentage of cerebral venules (56.5%) in the infected group presented PLAs at 15 dpi, compared with 5% at 8 dpi (*p*<0.05), while the NI control group (*p*<0.01) did not exhibit any microvascular PLAs (Figure 4C).

We measured malondialdehyde production in the brain of infected mice to assess changes in the local oxidative stress. Infected mice presented an increase in brain malondialdehyde production at 8 dpi, indicating increased oxidative stress (Figure 4E, *p*<0.01), which corresponded to the peak of parasitemia (Figure 1A) and to the initial increase in leukocyte rolling (Figure 4A). At 15 dpi, the production of malondialdehyde returned to the control group level (Figure 4E), which corresponded to the decrease in parasitemia.

### *T. cruzi* infection causes endothelial dysfunction in cerebral microcirculation

**Figure 5:**
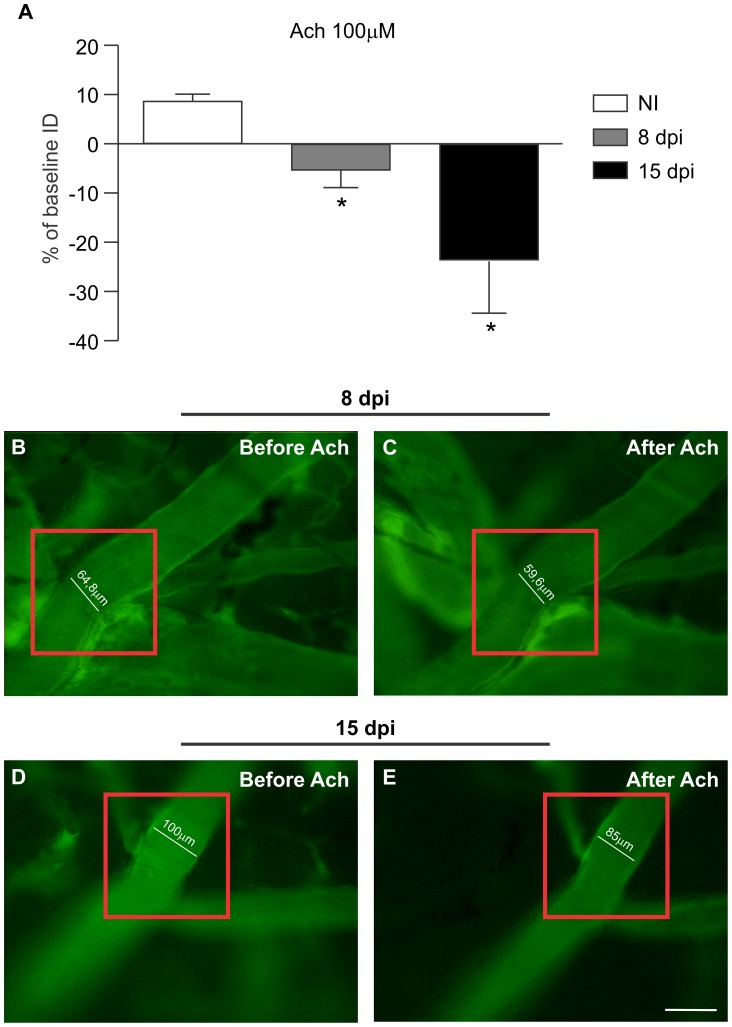
Acute CD causes endothelial dysfunction manifested as changes in vascular reactivity to acetylcholine. The graph shows a reduction in the vascular internal diameter (ID) after acetylcholine treatment in infected animals (A). In the NI group, the ID of cerebral arterioles increased 8.3±1.7% from the baseline value, whereas in the *T. cruzi*-infected mice, Ach induced a significant vasoconstrictor response of 5.4±3.3% at 8 dpi and of 23.10±11.2% at 15 dpi. (B–E): Representative images of cerebral arterioles of *T. cruzi*-infected mice showing a reduction in internal diameter after exposure to Ach at 8 (C) and 15 (E) dpi. Quantitative data are expressed as the means ± SEM (n = 5–8/group). Unpaired t test; *p*<0.05^*^; dpi: days post-infection; NI: non-infected.

We evaluated the endothelial function of cerebral arterioles after the topical application of Ach to the cranial window. The acute *T. cruzi* infection significantly impaired the endothelium-dependent vasodilatation induced by Ach. In the NI group, the inner diameter (ID) of cerebral arterioles increased 8.3±1.7% from the baseline values, indicating a preserved endothelial function. In *T. cruzi*-infected mice, Ach induced a marked vasoconstrictor response of 5.4±3.3% at 8 dpi and 23.10±11.2% at 15 dpi (*p*<0.05 compared with the NI group at 8 and 15 dpi; Figure 5A), which indicated cerebral endothelial dysfunction. Figure 5 presents representative images of cerebral arterioles before (Figures 5B and D) and after (Figures 5C and E) the application of Ach in infected animals at 8 and 15 dpi, showing that Ach induced vasoconstriction, reducing the arteriolar ID (Figures 5C and E).

## Discussion

In the present work, we showed for the first time that experimental acute CD increases oxidative stress in the brain and induces cerebral microvasculopathy in mice, suggesting the involvement of these alterations in the pathophysiology of CD. We utilized SWM, which are susceptible to the Y strain of *T. cruzi* when infected with trypomastigote forms of this parasite. Our results showed that the infected animals had a peak of parasitemia at 8 dpi, body weight loss starting at 12 dpi and high mortality (80%) at approximately 22 dpi; these results agree with observations of acute CD in previous studies [29], [30]. Using IM, we observed significant alterations in cerebral microcirculation, such as functional microvascular rarefaction, increased leukocyte rolling and adhesion, a high number of microvessels presenting PLAs and significant endothelial dysfunction.

Cerebral manifestations, such as meningoencephalitis, occur during the acute phase of CD, mainly in children or during the reactivation of the disease in immunosuppressed patients [4], [31]. Furthermore, cognitive impairment [6] and depressive behavior have also been associated with chronic CD in humans [7]. Moreover, in experimental models of acute and chronic CD in mice, an association between systemic inflammation and depressive-like behavior was observed [8]. Despite the association of ischemic stroke with chagasic cardiomyopathy [5], [32], ischemic stroke also occurs in *T. cruzi*-infected patients without left ventricle dysfunction. In addition, small-vessel disease occurs in these patients, supporting the idea that stroke subtypes, other than cerebral embolism of cardiac origin, should be considered in CD patients. This idea suggests an association of this manifestation with microcirculatory alterations [33].

The involvement of microcirculation in the pathophysiology of chagasic cardiomyopathy is well established [16]. Nevertheless, cerebral microvascular alterations in CD are much less studied but potentially damaging. Previous reports showed endothelial activation [22], [34] and the presence of *T. cruzi* nests in cerebral endothelial cells in experimental CD [23].

The advanced experimental method of the cranial window, which has been developed for intravital microscopy of the cerebral microcirculation, has been successfully used by different research teams, including our group. This technology allows the visualization of cerebral arterioles, venules and capillaries and has been used to evaluate functional capillary density [17], [26], [27], [35]. In the present study, we observed notable cerebral functional capillary rarefaction, verified by a reduction in the number of perfused capillaries at 15 dpi, indicating microcirculatory collapse in the brain of infected mice. Abnormal cardiac microcirculation with focal vascular constriction, microaneurysm formation, dilatation and microvessel proliferation has also been demonstrated in *T. cruzi*-acutely infected animals [36]. Moreover, *T. cruzi*-infected mice showed a significant decrease in the flow of red blood cells in arterioles and venules of the cremaster muscle [21].

Vasoactive substances are involved in the modulation of the vascular response during *T. cruzi* infection [16], [37]–[40]. Endothelin-1 is an endothelium-derived contracting factor [41] that participates in the microvascular dysfunction in CD [38] and is involved in the invasion of host cells by *T. cruzi* [20]. Recently, it has been demonstrated that the blockade of endothelin receptors increased the parasitemia and decreased the initial resistance of the central nervous system to *T. cruzi* infection in rats [42]. In this context, endothelin may be involved in the cerebral functional capillary rarefaction observed in the present study. Another vasoactive molecule considered to be a key regulator of CD pathogenesis is eicosanoid thromboxane. Thromboxane is a vasoconstrictor, promotes platelet aggregation, increases vascular permeability and is a potent pro-inflammatory molecule. High levels of thromboxane B_2_ and increased platelet aggregation were observed in *T. cruzi*-infected mice, suggesting that endothelial cell dysfunction and increased platelet reactivity could contribute to microvascular spasm and occlusion in acute CD [37]. Moreover, it was demonstrated that thromboxane A_2_, produced by *T. cruzi*, accounts for most of the circulating thromboxane in infected animals [40]. Therefore, thromboxane may be implicated in the high occurrence of PLAs in cerebral microvessels observed in this study at 15 dpi, which could contribute to functional capillary rarefaction in the brains of animals.

We also investigated the leukocyte-endothelium interaction in cerebral microcirculation. We observed a high number of leukocytes rolling and adhering to the cerebral venules of infected mice. At 8 dpi, there was already a significant increase in the number of rolling leukocytes, and this increase was even greater at 15 dpi. Leukocyte adhesion was quite pronounced at 15 dpi and correlated with the high number of inflammatory cells in the heart at the same time post-infection (data not shown). Previous studies demonstrated the activation of the cerebral vascular endothelium by an increase in the expression of VCAM-1 in experimental acute CD and in immunosuppressed, chronically infected animals. In addition, the role of the VLA-4/VCAM-1 pathway in the establishment of meningoencephalitis induced by *T. cruzi* has been suggested [22], [34]. Although we did not characterized the profile of the inflammatory cells, it was previously demonstrated that during experimental meningoencephalitis induced by *T. cruzi*, the infiltrating cerebral lymphocytes in the brain consisted mainly of CD8^+^ T cells [34].

*T. cruzi* induces an increase in the production of reactive oxygen species (ROS) in cardiomyocytes, which is enhanced by IL-1β, TNF-α and IFN-γ [43]. Moreover, the mitochondrial generation of ROS was observed in the myocardium of *T. cruzi*-infected mice [44] and of patients with CD [45]. Here, we evaluated the production of malondialdehyde, a marker of lipid peroxidation in the brain of mice, as an indirect measure of oxidative stress [46]. We found an increase in the production of malondialdehyde in the brain of infected animals at 8 dpi, which corresponds to the parasitemia peak. Because high parasitemia is associated with systemic inflammation [47], our data suggest that oxidative damage could be associated with an initial inflammatory response to the parasite, which could lead to an increase in pro-inflammatory cytokines and chemokines in the blood, endothelial and inflammatory cell activation and the release of ROS in the brain during acute CD. The NADPH-oxidase system is a source of ROS in phagocytic cells, and malondialdehyde production can enhance the activity of the system, especially during the response to pathogens [48]. Furthermore, a recent study showed that oxidative stress contributes to the persistence of *T. cruzi* in mouse tissues [49].

The inflamed pro-thrombotic endothelium and the excess oxidative excess observed in the present study in the brain of *T. cruzi*-infected mice have also been known to be involved in the reduced vascular reactivity in other pathologies [50]. Therefore, we investigated cerebral endothelial function. Our results showed a severe alteration in the microvascular reactivity to acetylcholine stimulation in infected animals, characterized by the vasoconstriction of arterioles, suggesting damage to the cerebral microvascular endothelial layer. It is well known that the injury of endothelial cells can result in the direct action of acetylcholine on smooth muscle cells of the mural layer, resulting in arteriole contractions [51]. In fact, in the presence of endothelial dysfunction, muscarinic agonists produce vasoconstriction by direct M3 receptor activation [52], [53]. Endothelial dysfunction was present at 8 dpi, when the cerebral oxidative stress increased and the leukocyte-endothelium interaction began, as observed by an increase in the number of rolling leukocytes. The endothelial dysfunction persisted to 15 dpi, concurrently with an increased number of adherent inflammatory cells, microvascular PLAs and cerebral capillary rarefaction. The NI mice showed a preserved endothelial-dependent response that was characterized by vascular dilatation in response to acetylcholine, suggesting the release of nitric oxide by endothelial cells after stimulation [54]. These results indicate that increased oxidative stress and the augmented leukocyte-endothelium interaction contribute to cerebral microvascular endothelial dysfunction and the reduction of functional capillary density in *T. cruzi*-infected animals. Our findings corroborate the results of previous studies that also observed alterations in the mechanisms involved in the regulation of vascular function in CD. Patients with CD without heart failure presented venous endothelial dysfunction in response to acetylcholine [55]. In another study, flow-mediated, endothelium-dependent vasodilatation and nitroglycerin-mediated vasodilatation of the humeral artery were evaluated in patients with chagasic cardiomyopathy. There were no differences in flow-mediated vasodilatation. However, the activity of nitroglycerin, which induces endothelium-independent vasodilatation, was lower in the patients, suggesting a dysfunction of vascular smooth muscle cells [56].

In conclusion, the results of the present study demonstrate that acute CD causes significant cerebral microvasculopathy as well as increased cerebral oxidative stress in mice. An acute *T. cruzi* infection leads to functional capillary rarefaction, an increase in rolling and adhered leukocytes, microvascular PLA formation and noticeable endothelial dysfunction in the cerebral microcirculation. Furthermore, our data support the idea that these cerebral microcirculatory changes may result in long-term consequences, contributing to neurological manifestations of chronic CD. Finally, the mechanisms involved in cerebral microvascular alterations and increased cerebral oxidative stress could be further investigated as novel therapeutic targets for the treatment of CD.
