# Correction: Acute Chagas Disease Induces Cerebral Microvasculopathy in Mice

**DOI:** 10.1371/journal.pntd.0003151

**Published:** 2014-08-13

**Authors:** The PLOS Neglected Tropical Diseases Staff

In Figure 1, the axis for Part C is incorrect. The authors have provided a corrected version here.

**Figure 1:**
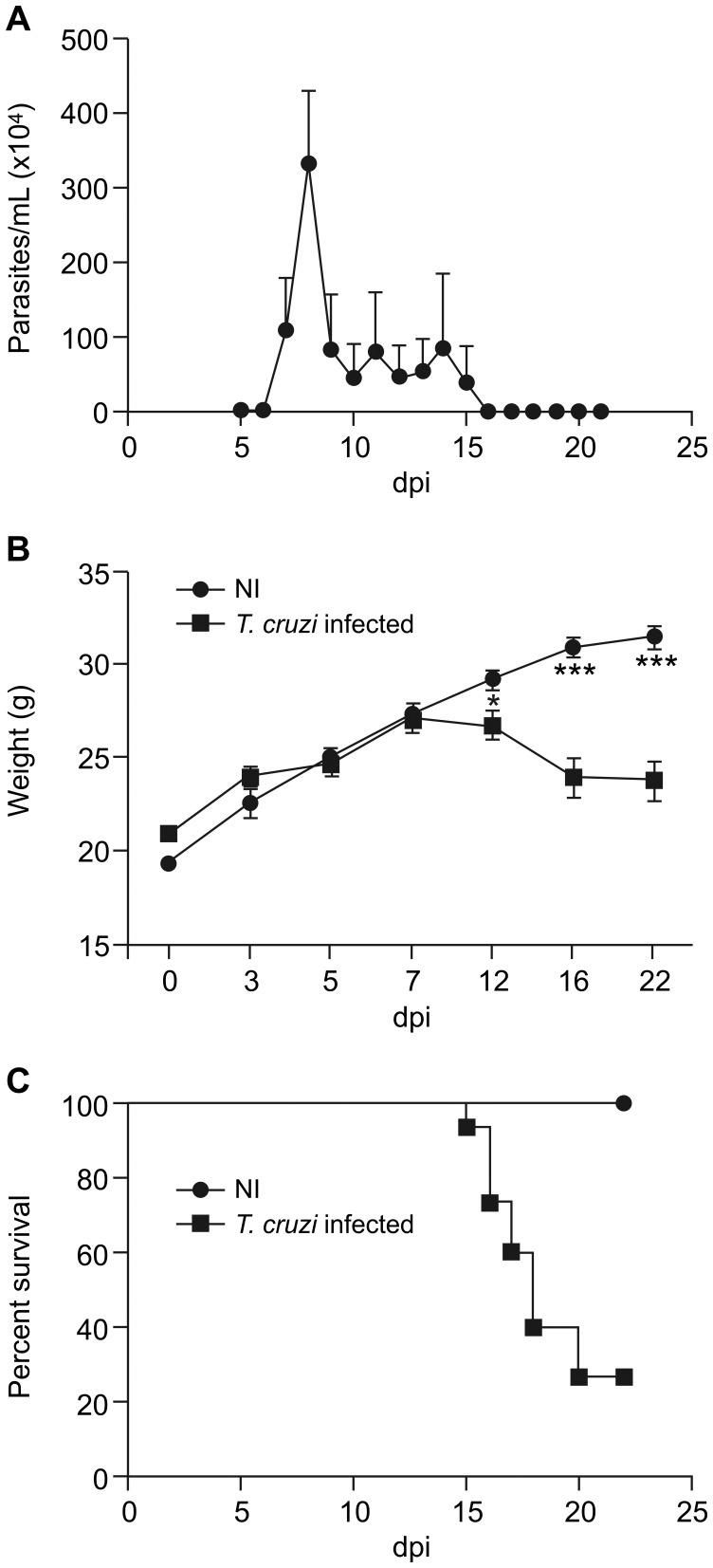
Swiss Webster mice infected with the Y strain of *T. cruzi*-developed acute CD. Mice were infected with 10^4^ blood trypomastigote forms, and the following parameters were evaluated in a kinetic study: (A) parasitemia, (B) weight and (C) survival rate. The parasitemia peak occurred at 8 dpi (A). *T. cruzi* infection induced a significant body weight decrease in a time-dependent manner, starting at 12 dpi. At 22 dpi, the average weight of the NI mice was 31.4±1.9 g, while that of the *T. cruzi*-infected group was 23.8±2.5 g (B). At 22 dpi, only 20% of the infected animals survived (C). Quantitative data are expressed as the means ± SEM (n  =  20). One-way ANOVA test, *p*<0.05^*^ and *p*<0.001^***^, comparing the infected group at 15 dpi with the NI group; dpi: days post-infection; NI: non-infected.
